# A Rare Cause of an Acute Myocardial Infarction in a Young Pregnant Woman After Misoprostol-Induced Abortion

**DOI:** 10.7759/cureus.90655

**Published:** 2025-08-21

**Authors:** Mohammad A Motaweih, Bhavana Baraskar, Yongdeok B Shin, Micaela Iantorno

**Affiliations:** 1 Internal Medicine, Mary Washington Hospital, Fredericksburg, USA; 2 Cardiology, Mary Washington Hospital, Fredericksburg, USA

**Keywords:** postpartum scad, rare scad case requiring intervention, scad in pregnancy, scad management, scad types

## Abstract

Spontaneous coronary artery dissection (SCAD) is an uncommon but increasingly recognized cause of acute coronary syndrome (ACS), myocardial infarction (MI), and sudden cardiac death, particularly in younger individuals without traditional cardiovascular risk factors. Pregnancy-associated SCAD (P-SCAD) is a specific subset of SCAD that accounts for a significant number of MIs in pregnant and postpartum women. Misoprostol is a prostaglandin E1 analogue that is used for medical termination of pregnancy and has been associated with coronary vasospasm in rare cases; however, our case highlights a potential new association between this drug and SCAD.

A 29-year-old African American woman with no significant past medical history presented to the emergency room with seizure-like activity and unresponsiveness witnessed by her sister while they were traveling on a road trip few hours after taking Misoprostol for termination of an ectopic pregnancy. Coronary angiography showed focal 95% stenosis of the proximal left anterior descending (LAD) artery with TIMI 1 flow concerning spontaneous coronary artery dissection versus coronary vasospasm. A decision was made to proceed with percutaneous coronary intervention to the LAD. The procedure was complicated by no reflow and retrograde advancement of dissection/hematoma to the left main coronary artery, requiring further stenting with overlapping stents from the ostial left main coronary artery to the proximal LAD artery.

During the procedure, the patient developed multiple cardiac arrests with pulseless electrical activity requiring advanced cardiac life support, pressors, and left ventricular assist device (LVAD) placement for mechanical circulatory support. She subsequently showed marked improvement and was successfully weaned off vasopressors, and the LVAD was removed by day 4 in the ICU. On day 8, the patient was successfully extubated. The follow-up 2D echocardiogram showed improvement in the ejection fraction to 40-45%. The patient was safely discharged and made significant cardiovascular recovery.

This is an interesting case discussion highlighting a rare and potential association between a commonly prescribed medication and a serious cardiovascular complication. It has been observed that SCAD occurs with higher frequency during pregnancy, peripartum period, and with the use of exogenous hormones although the exact etiology remains unclear. More research should be done to better understand the potential cardiovascular adverse effects of Misoprostol, especially on patients with underlying SCAD.

## Introduction

Spontaneous coronary artery dissection (SCAD) is an uncommon but increasingly recognized cause of acute coronary syndrome (ACS), myocardial infarction (MI), and sudden cardiac death, particularly in younger individuals without traditional cardiovascular risk factors [1]. Unlike atherosclerotic MI, SCAD results from a tear or separation in the coronary arterial wall, leading to blood flow obstruction and ischemia. Despite its rarity, SCAD can often go undetected or be misdiagnosed, emphasizing the need for greater clinical awareness [1-3].

As noted by Stanojevic et al., SCAD is linked to several precipitating factors, including hormonal changes, connective tissue disorders, and extreme emotional or physical stress. In the context of pregnancy-associated SCAD (P-SCAD), the most consistently implicated risk factors are elevated hormonal levels, particularly estrogen and progesterone, which contribute to weakened collagen structure and impaired vascular integrity, as well as hemodynamic stress from increased cardiac output and volume shifts during and after pregnancy [1].

P-SCAD is a specific subset of SCAD that accounts for approximately 43% of MIs in pregnant and postpartum women under age 50, with the highest incidence occurring during the early postpartum period [4]. While rare, it is a critical diagnosis to consider given its association with significant morbidity and mortality [5-8].

Misoprostol, a prostaglandin E1 analogue widely used for medical termination of pregnancy, has been implicated in reports of rare cases of coronary vasospasm. Although no direct association between Misoprostol and SCAD has been established in the literature, coronary vasospasm could theoretically contribute to SCAD pathogenesis. Intense vasospasm may result in transient ischemia and abrupt changes in shear stress, potentially triggering intimal injury or promoting the development of intramural hematoma, both of which are proposed mechanisms in SCAD [5-9].

This report discusses a rare presentation of P-SCAD occurring in the first trimester following medically induced abortion with Misoprostol. The case underscores the complex interplay between pregnancy-related hormonal and hemodynamic changes and potential iatrogenic factors. It also highlights the need for further research to clarify the mechanistic pathways linking hormonal therapies, such as Misoprostol, to vascular complications, including SCAD.

## Case presentation

**Figure 1:**
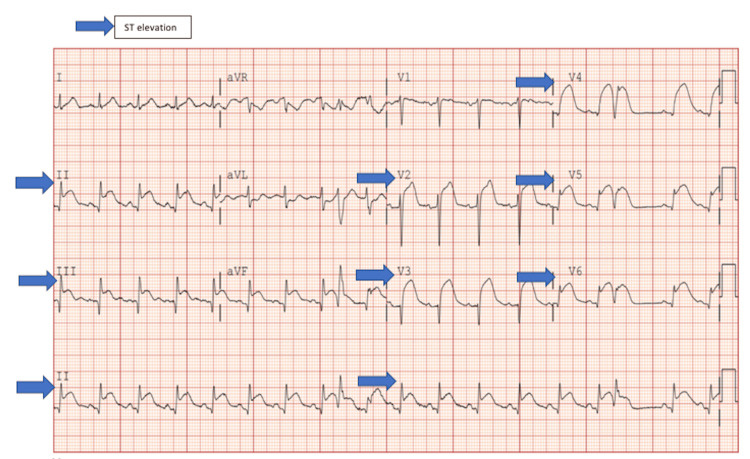
EKG showing diffuse ST segment elevation in leads II, III, aVF, and v2-v6.

A 29-year-old African American woman with no significant past medical history presented to the emergency room with seizure-like activity and unresponsiveness witnessed by her sister while they were traveling on a road trip. Vital signs on presentation were: T 99.4°F, HR 104 b/m, BP 106/73 mmHg, and SpO2 93% on room air. The patient had received the second dose of Misoprostol for termination of an ectopic pregnancy a few hours prior to the event. The patient denied the use of any other hormonal medications or having known allergies; there was no significant history of cardiac disease in the family. The patient reported pack a day smoking but has no alcohol or illicit drug use history. On exam patient was lethargic and was not able to provide a review of systems. In the emergency department, initial CT head without contrast showed no acute intracranial pathology. Her electrocardiogram (EKG) showed diffuse ST-segment elevations in leads II, III, AVF, and V2-V6 (Figure 1).

**Figure 2:**
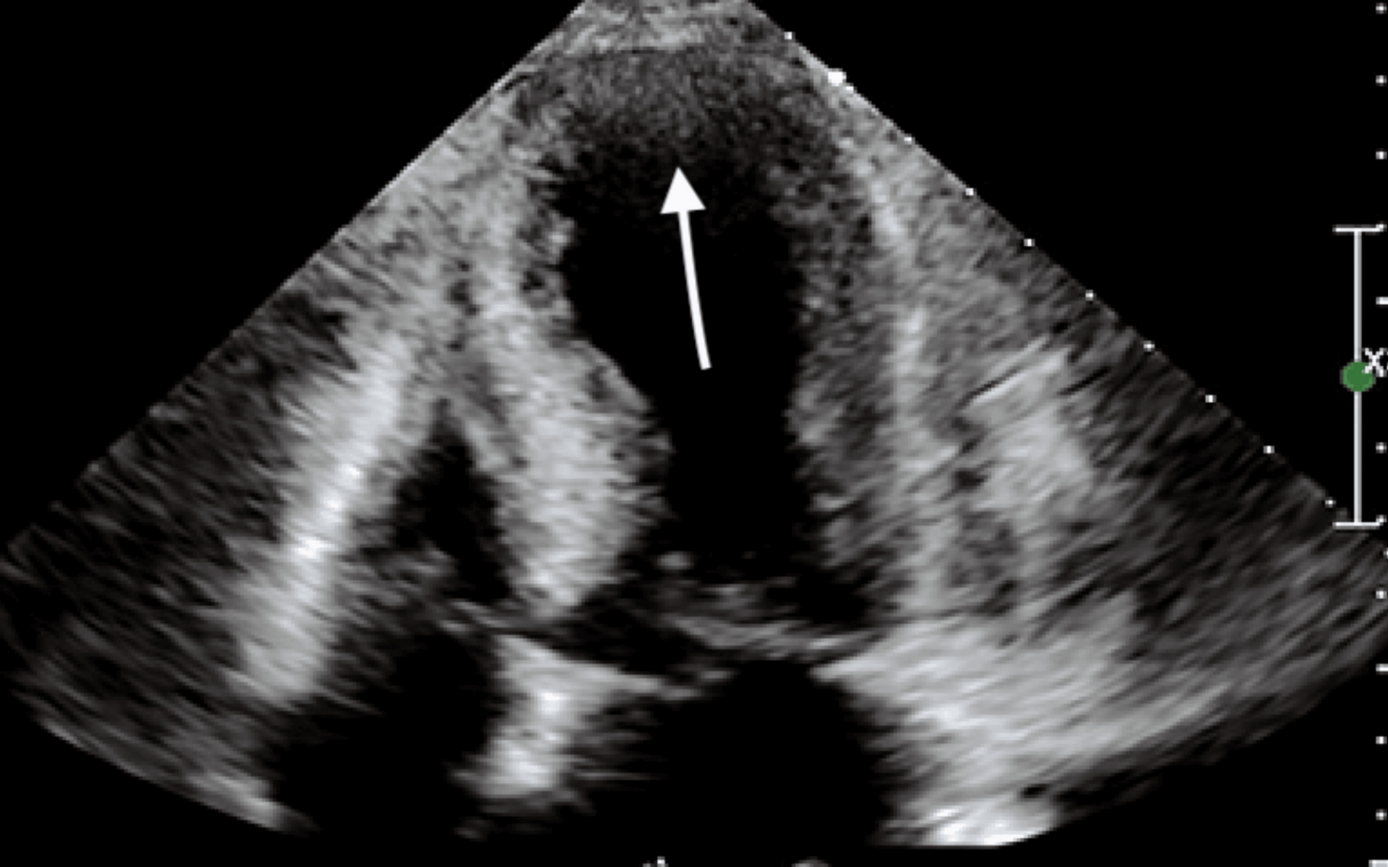
Echo image showing apical wall hypokinesia during systole.

Labs were remarkable for elevated troponin of 0.139 ng/ml (reference range: 0-0.04ng/ml), positive urine pregnancy test, and potassium of 3 mmol/L. Initial 2D echocardiogram revealed left ventricular systolic dysfunction with an ejection fraction (EF) of 35-40% and severe apical wall hypokinesis (Figure 2).

**Figure 3:**
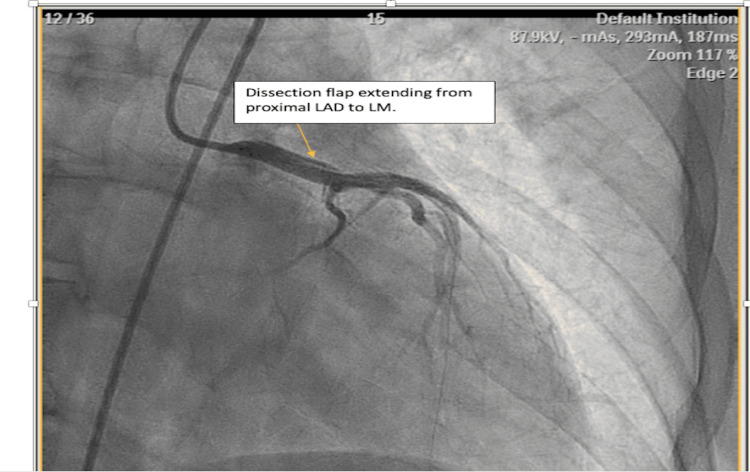
Coronary angiogram image showing retrograde dissection extending into the ostial left main coronary artery

Subsequently, the patient was taken to the Cath-lab, where coronary angiography showed focal 95% stenosis of the proximal left anterior descending (LAD) artery with TIMI 1 flow concerning for spontaneous coronary artery dissection. A decision was made to proceed with percutaneous coronary intervention (PCI) to the LAD artery. The procedure was complicated by retrograde advancement of dissection/hematoma to the left main coronary artery requiring further stenting with overlapping stents from the ostial left main coronary artery to the proximal LAD (Figure 3).

**Figure 4:**
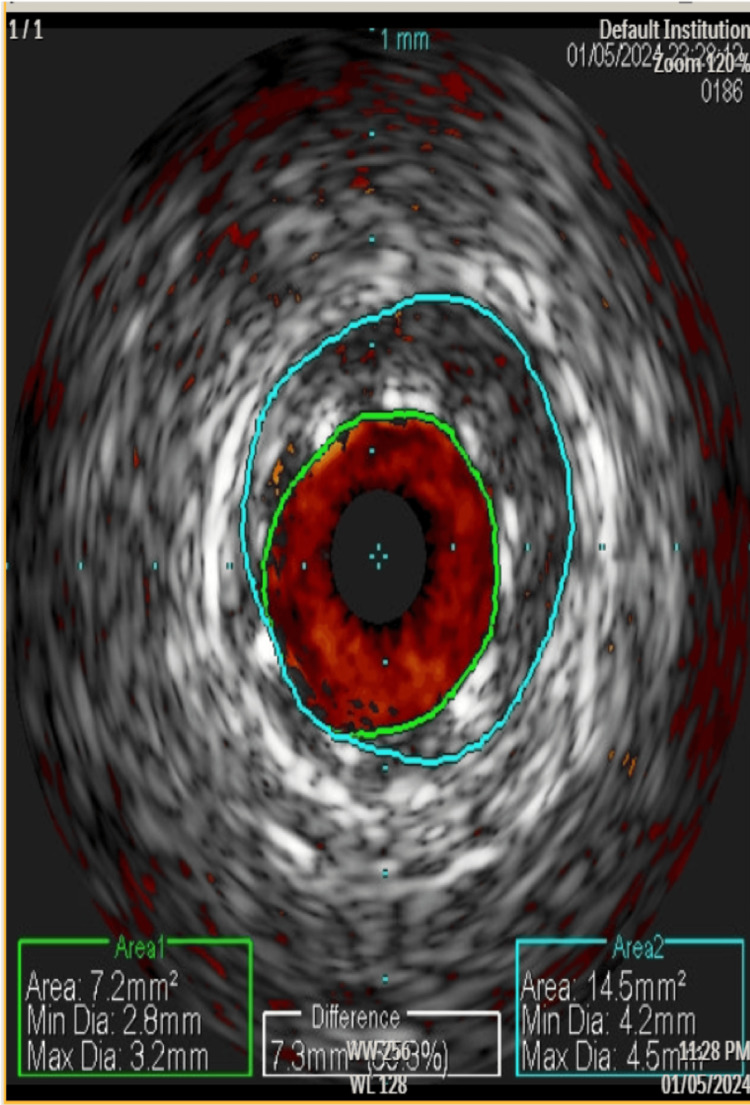
IVUS image post stenting showing possible dissection flap IVUS: Intravascular ultrasound

Post-stenting intravascular ultrasound (IVUS) imaging was obtained which showed the dissection flap suggesting that SCAD is the more likely diagnosis (Figure 4).

**Figure 5:**
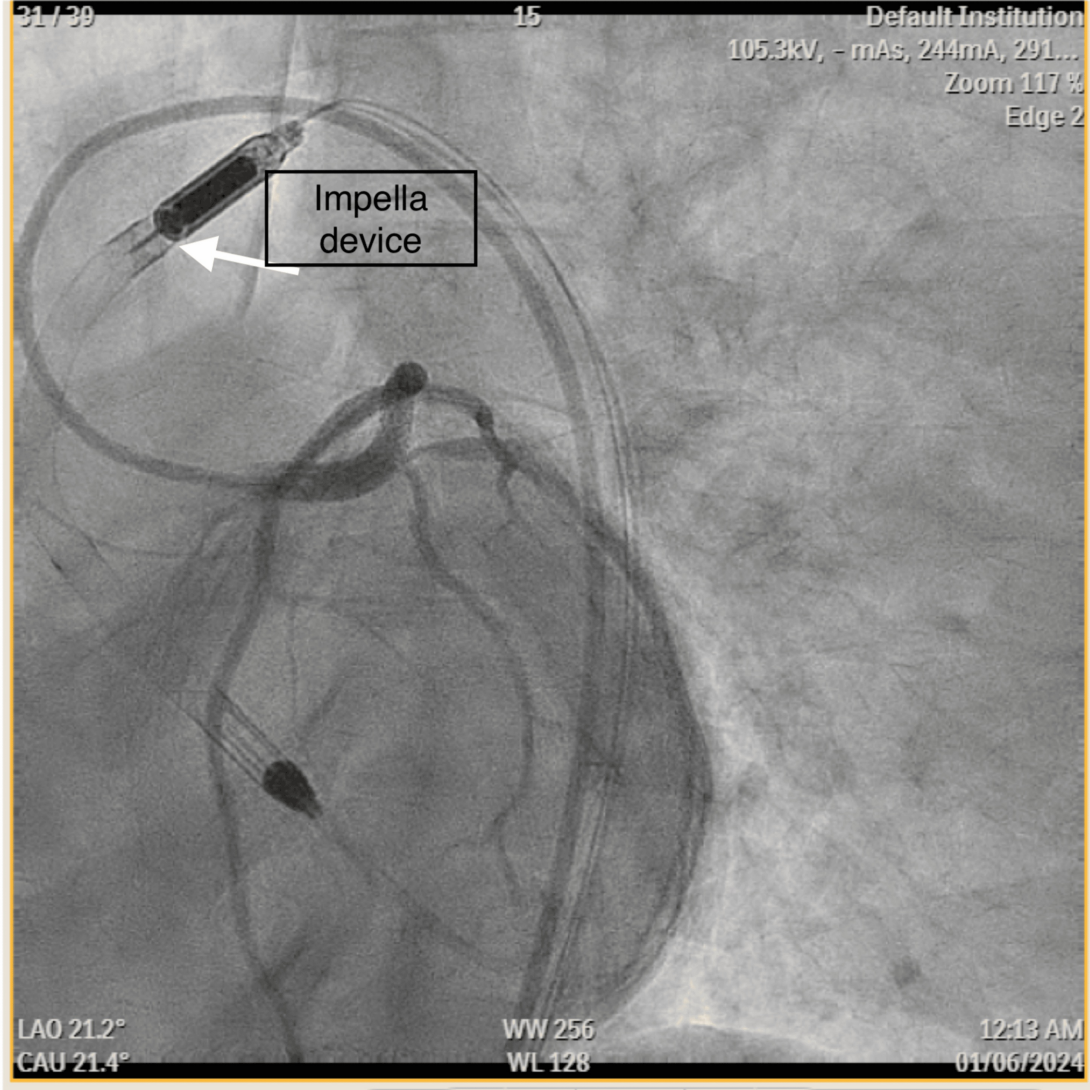
Coronary angiography image showing results post-PCI and left ventricular mechanical assist device insertion. PCI: Percutaneous coronary intervention

During the procedure, the patient developed multiple cardiac arrests with pulseless electrical activity requiring advanced cardiac life support, later followed by initiation of vasopressors and emergent intubation. Additionally, an Impella device was placed for left ventricular support and cardiogenic shock, later followed by transfer to the intensive care unit (ICU) (Figure 5).

The patient was started on aspirin and Brilinta. Over the course of the next few days, she showed marked improvement, regaining hemodynamic stability. She was weaned off vasopressors and Impella by day 4 in the ICU. The course in the ICU was complicated by pulmonary edema requiring IV diuresis. On day 8, the patient was successfully extubated. The follow-up 2D echocardiogram showed improvement in the ejection fraction to 40-45%. An extensive workup was done to rule out autoimmune vasculitis and connective tissue disorders, including ANCA vasculitis panel, ANA, and RF. The patient was later safely transferred to the medical floor and was subsequently discharged on guideline-directed medical therapy (GDMT). The patient was instructed to follow up in the outpatient clinic closely to optimize GDMT as well as further workup for potential underlying genetic abnormalities like fibromuscular dysplasia.

## Discussion

SCAD was once considered a rare cause of non-atherosclerotic coronary artery disease. However, it is now increasingly recognized, particularly among young and middle-aged women without traditional cardiovascular risk factors. According to prospective registries, SCAD is overwhelmingly reported in women (female-to-male ratio of 9:1), accounting for up to 35% of myocardial infarctions in women aged ≤50 years and up to 43% of pregnancy-associated myocardial infarctions (P-SCAD) [1-9].

P-SCAD more frequently involves the LAD and left main (LM) coronary arteries and is often more diffuse or multivessel in nature, leading to a higher risk of reduced left ventricular function and life-threatening complications [4]. Clinicians should maintain a high index of suspicion for SCAD in women presenting with chest pain following a recent pregnancy or abortion.

The pathophysiology of SCAD remains incompletely understood, but two main mechanisms have been proposed. The “inside-out” hypothesis involves an intimal tear that allows blood to enter the arterial wall, creating a false lumen. In contrast, the “outside-in” mechanism suggests spontaneous hemorrhage from the vasa vasorum leads to intramural hematoma formation, which compresses the true lumen and restricts blood flow [5].

Pregnancy-related hormonal changes are believed to increase vascular vulnerability. Elevated progesterone may reduce collagen synthesis and promote fragmentation of elastic fibers, while estrogen can contribute to a hypercoagulable state and cystic medial necrosis [6-8]. Together, these effects may weaken the arterial wall and predispose to dissection under stress.

Although Misoprostol has been associated with coronary vasospasm in rare case reports, there is currently no observational or mechanistic evidence linking it directly to SCAD. Coronary vasospasm could theoretically increase shear stress or promote intramural bleeding, potentially triggering SCAD in predisposed individuals; however, this remains speculative. Caution is warranted before attributing causality without further data.

SCAD is angiographically classified using the Yip-Saw classification system, which delineates three main types based on coronary angiographic appearance. Type 1 SCAD is the most pathognomonic and is characterized by contrast dye entering and separating multiple lumens, producing the classic appearance of radiolucent flaps within the coronary artery. Type 2 SCAD is the most commonly observed angiographic form and presents as a long, smooth, tubular narrowing, typically due to intramural hematoma without an evident intimal tear. Type 2 is further subdivided: Type 2a lesions show distal restoration of the normal vessel caliber, whereas Type 2b lesions extend distally to the terminal end of the vessel without visible recovery of lumen size. Type 3 SCAD mimics atherosclerotic stenosis and typically presents as a focal, discrete narrowing of the vessel. Because Type 3 lesions are angiographically indistinct from typical plaque-related disease, intravascular imaging modalities such as OCT (optical coherence tomography) or intravascular ultrasound are often required for definitive diagnosis [3].

Prompt diagnosis is essential to guide appropriate management. Coronary angiography remains the gold standard for diagnosis. Thrombolytics are generally contraindicated due to the risk of extending the dissection or worsening intramural hematoma. Conservative medical therapy is preferred in stable patients, as PCI has been associated with increased risk of iatrogenic dissection and abrupt vessel closure [10-13].

Invasive strategies, including PCI or mechanical circulatory support (MCS) such as Impella or veno-arterial extracorporeal membrane oxygenation (VA-ECMO), are reserved for unstable patients with ongoing ischemia or cardiogenic shock [14,15]. Medical management should be tailored to the individual. Long-term aspirin is typically continued due to its favorable safety profile. The benefit of adding clopidogrel (dual antiplatelet therapy) in patients who have not undergone PCI remains uncertain and should be considered on a case-by-case basis. Statins are not routinely indicated unless the patient has concomitant dyslipidemia or atherosclerotic disease.

Follow-up care is critical in the SCAD population. SCAD has a recurrence rate of up to 10.4%, as shown in the Canadian cohort study [13]. Patients should be counseled on the risk of recurrence, the importance of blood pressure control, stress management, and the need to avoid extreme physical exertion. Participation in cardiac rehabilitation is encouraged. Women should also receive individualized counseling regarding future pregnancy, contraception, and psychosocial support.

In our patient’s case, given her hemodynamic instability, an invasive approach was deemed necessary. SCAD management should be individualized based on clinical severity and stability. Randomized controlled trials are lacking, and current treatment approaches are based on observational studies and expert consensus. PCI and coronary artery bypass grafting (CABG) should be reserved for high-risk patients where potential benefits outweigh procedural risks.

Our case is notable for a rare presentation of SCAD following medically induced abortion using Misoprostol. Although a temporal association exists, there is no definitive evidence linking Misoprostol to SCAD. We identified a prior case in the literature involving Misoprostol-associated coronary vasospasm and cardiac arrest [16], highlighting the need for further research into the cardiovascular effects of pharmacologic agents used in reproductive care.

Impella is a percutaneous short-term MCS device used to provide hemodynamic support in a range of scenarios, from high-risk PCI to cardiogenic shock [3]. In our patient, the use of Impella was crucial in stabilizing her hemodynamics and achieving a favorable outcome. Cardiogenic shock occurs in approximately 2-19% of SCAD cases, with a higher incidence in patients presenting with ST-elevation myocardial infarction [2,3].

In conclusion, long-term monitoring and structured patient counseling are essential components of SCAD care. As SCAD can recur and carries significant emotional and physical consequences, multidisciplinary follow-up is necessary to support recovery and reduce recurrence risk.

## Conclusions

SCAD treatment should be individualized based on clinical severity and hemodynamic stability. Given the lack of randomized controlled trials, current management is guided primarily by observational data and expert consensus. Revascularization strategies such as PCI or CABG should be reserved for high-risk scenarios, typically defined as patients with ongoing or refractory ischemia, left main or proximal multivessel involvement, hemodynamic instability, or cardiogenic shock. These interventions carry elevated procedural risks in SCAD, including extension of dissection and iatrogenic injury, and are best considered when the anticipated benefit outweighs the potential harm.

In contrast to most SCAD presentations, our case represents a unique instance of SCAD following medically induced abortion with Misoprostol. While a direct causal link remains unproven, the temporal association highlights the need for further investigation into SCAD pathogenesis and its possible relationship with pharmacologic agents used in reproductive care. Long-term monitoring is essential, and patients require structured follow-up, risk stratification, and individualized counseling. Further research is urgently needed to elucidate underlying mechanisms, identify vulnerable populations, and guide the development of evidence-based treatment and prevention strategies.
